# Biochemistry of Bacterial Multidrug Efflux Pumps

**DOI:** 10.3390/ijms13044484

**Published:** 2012-04-10

**Authors:** Sanath Kumar, Manuel F. Varela

**Affiliations:** 1 QC Laboratory, Post Harvest Technology Department, Central Institute of Fisheries Education (CIFE), Versova, Mumbai 400061 India; E-Mail: sanathkumar@cife.edu.in; 2 Biology Department, Eastern New Mexico University, Portales New Mexico, 88130, USA

**Keywords:** efflux pump, antibiotic resistance, MFS, antiporter, Tet, Qac

## Abstract

Bacterial pathogens that are multi-drug resistant compromise the effectiveness of treatment when they are the causative agents of infectious disease. These multi-drug resistance mechanisms allow bacteria to survive in the presence of clinically useful antimicrobial agents, thus reducing the efficacy of chemotherapy towards infectious disease. Importantly, active multi-drug efflux is a major mechanism for bacterial pathogen drug resistance. Therefore, because of their overwhelming presence in bacterial pathogens, these active multi-drug efflux mechanisms remain a major area of intense study, so that ultimately measures may be discovered to inhibit these active multi-drug efflux pumps.

## 1. Introduction: Antimicrobial Resistance and Drug Efflux Pumps

Morbidity and mortality rates caused by infectious microbial agents represent serious public health concerns. Pathogenic bacteria are causative agents of infectious disease in all body systems and are particularly serious due to their consequences economically, socially, and in regards to quality of life [1–3]. Thus, it is critical that a clear understanding of the biological aspects of infectious disease be made known so that eventually infectious disease morbidity and mortality are curtailed, if not eventually eradicated. Pathogenic bacteria have devised virulence factors, such as drug resistance determinants, that facilitate pathogenesis in human patients. Unfortunately, many of these bacterial virulence factors are poorly understood at the molecular level. Thus, it is critical that such virulence factors be understood mechanistically so that they may be used as targets for chemotherapy. Antimicrobial agents are indicated for bacterial infectious disease treatment and represent a good means of combating infectious disease [4]. Regrettably, many pathogenic bacteria have acquired or developed resistance mechanisms, which work against anti-bacterial drugs [5,6]. Further, use and abuse of individual antimicrobial agents in clinical settings have selected for variant bacterial pathogens that are naturally resistant to anti-bacterial drugs [7]. In fact, selection of single-drug resistance often has led to selection of multi-drug resistance in pathogenic bacteria [8]. Such multi-drug resistant bacterial pathogens compromise chemotherapeutic efforts and enhance both morbidity and mortality rates in humans [5,6]. Therefore, multi-drug resistant mechanisms represent extremely good targets for studies towards the efforts in the effective treatment of infectious disease [9,10]. Among various reasons for the development of antibiotic resistance in bacteria, overuse is considered the most important, though this does not explain the presence of antibiotic resistance in non-pathogenic environmental bacteria which are not exposed to antibiotics [11]. Many species of bacteria are now known to have armed themselves with the means of fighting toxic compounds in nature, and such abilities have manifested as antibiotic resistance mechanisms in human pathogenic bacteria. While the evolution of antibiotic resistance in response to the toxic compounds is straightforward and easy to comprehend, bacteria on the other hand, possess complex machineries, which extrude antibiotics as their secondary function. These membrane bound multidrug resistance (MDR) efflux pumps are found in all bacteria and their primary functions could be other than antibiotic resistance, which include maintenance of intracellular solute concentrations or the cell homeostasis, extrusion of toxic byproducts of metabolism or transport of amino acids and nucleotides [12–15]. Efflux pump-mediated resistance to single or multiple antimicrobial agents has not only raised serious concerns but also has constricted the treatment options against bacterial infections [13]. Efflux pumps reduce the accumulation of antibiotics inside of the bacterial cells, and the slow phase in which the process of antibiotic efflux takes place provides sufficient time for the bacterium to adapt to the antibiotics and become resistant through mutations or alteration of antibiotic targets [16]. Further, the over-expression of efflux pumps enhances the resistances to antimicrobials [17]. Based on the sequence comparison, efflux pumps are grouped into five major categories: The major facilitator superfamily (MFS), the adenosine triphosphate (ATP)-binding cassette (ABC) family, the resistance-nodulation-division (RND) family, the small multidrug resistance (SMR) family and the multidrug and toxic compound extrusion family [18–27]. Efflux pumps are now known to confer resistance to almost all classes of antibiotics [19].

This review is focused mainly on efflux pumps of the MFS from Gram-negative and Gram-positive bacteria, current trends, and the future prospects for understanding the structure-functions relationships in drug efflux proteins.

## 2. Efflux of Tetracycline by TetA, A Key Drug Efflux Pump

Active efflux of antimicrobial agents was first discovered by Levy in which active extrusion was demonstrated of the tetracyclines from bacterial host cells harboring plasmid pBR322 [28,29]. Bacteria are able to resist the tetracyclines by way of active efflux *via* the TetA family of efflux pumps [30]. The TetA family of efflux pumps are grouped into two major groups: The first group is comprised of chromosomally-encoded 12-TMS efflux pumps, such as Tet(A), Tet(B), Tet(C) and Tet(D) [30–34], found in Gram-negative bacteria, while the second group comprises plasmid-encoded Tet(K) *Staphylococcus aureus* and Tet(L) found in *Bacillus* spp., *Staphylococcus*, and *Streptococcus* spp. [35]. Tet(K) and Tet(L) are 14-TMS efflux pumps [36,37]. Early work by Griffith and Henderson discovered shared homology between mammalian and bacterial sugar transporters [38,39], establishing the presence of the very large major facilitator superfamily of related symporters and antiporters with single- and multiple-drug substrates [24,40]. It was of tremendous interest that single- and multi-drug efflux pumps were homologous [24,41–44]. Implicit in this work was the presence of highly conserved amino acid sequence motifs shared in members of the MFS [24,27]. Later, a mutational analysis showed that a highly conserved glycine residue in the so-called antiporter motif (Motif C) of the TetA(C) efflux pump was necessary for conferring resistance to tetracycline [32]. This glycine was found in Motif C, a highly conserved motif in TMS5 in antiporters of the MFS [40]. Additional elements of Motif C were shown to be required for single and multidrug transport in the efflux pumps CaMdr1p, QacA, Mdt(A) from *Lactococcus garvieae* and *Lactococcus lactis*, Tet(B), Tet(K), Tet(L), and VAChT [32,45–54]. Inhibitors of tetracycline efflux were discovered [55–57]. Analyses of the structure-function relationships of single- and multi-drug efflux pumps may indentify key residues for discovery of efflux inhibitors. Therefore, identification of functionally conserved regions within efflux pumps should be useful for design of efflux pump inhibitors [58,59].

## 3. MdfA from *Escherichia coli*

MdfA, found in *Escherichia coli*, is a secondary multidrug efflux pump made up of 410 amino acids encoded by a chromosomal gene *cmr* [60]. Based on *phoA* (alkaline phosphatase) and *cat* (chloramphenicol acetyl transferase) gene fusion studies, MdfA has 12 transmembrane helices [61]. Though a crystal structure is not currently available, predicted 3D structure has been able to reveal some interesting features of MdfA such as the presence of a large cavity with a putative substrate binding function with three amino acids Glu26, Asp34 and Asp132 with critical roles in the interaction of MdfA with the drugs [62]. Of these, Glu26 has been shown to be important for the transport of cationic substrates [60]. Mutations changing Glu26 into Ala, Asn, His, Leu, and Asp severely affects the efflux of neutral substrates, while the amino acids Gln26 and Ile26 confer higher levels of resistance by MdfA to the same substrates [61]. On the other hand, a Glu26Thr mutation completely abolishes cationic drug transport by MdfA, and this effect is greatly reverted by a second site mutation Val335Glu/Asp [61]. In addition to multidrug resistance, MdfA performs a further function of maintaining the physiological pH of the cell [62].

## 4. EmrD-3 from *Vibrio cholerae*

Many of the antibiotic efflux pumps identified in the pathogenic *V. cholerae* belong to the MATE and RND family of efflux proteins [63–65]. EmrD-3, identified in an O1 strain of *V. cholerae*, is an efflux pump of MFS family with 12 transmembrane segments (TMS) [66]. Membrane proteins homologues of EmrD-3 are widely distributed among the Gram-positive and -negative bacteria, including several *Vibrio* and *Bacillus* spp. EmrD-3 is closely related to the Bcr/CflA subfamily of membrane proteins, which includes Bcr (bicyclomycin resistance protein) in *E. coli*, FloR (chloramphenicol and florfenicol resistance), in *Salmonella enterica* serotype Typhimurium DT104 and CmlA (chloramphenicol resistance) in *Pseudomonas*. The H^+^-antiport activity of EmrD-3 has been demonstrated by ethidium bromide efflux and accumulation assays [66]. Among the various antimicrobials actively extruded by EmrD-3 are linezolid, rifampin, trimethorprim, erythromycin, and chloramphenicol. Of these, the highest resistance was conferred to the oxzolidinone drug linezolid and it is speculated that linezolid may be the preferred efflux substrate by EmrD-3. The ability of EmrD-3 to actively extrude linezolid and the presence of its homologues in Gram-positive bacteria raises new concerns since the oxazolidinone class of drugs, to which linezolid belongs, are used to treat Gram-positive bacterial infections by *Streptococcus* spp., vancomycin-resistant *Enterococcus faecium*, and methicillin-resistant *Staphylococcus aureus* (MRSA) [67]. Resistance to linezolid is attributed to a point mutation in the peptidyl transferase of 23S rRNA and the involvement of efflux pumps is not well known [68]. Efflux pumps, being integral membrane proteins, were thought to extrude only hydrophobic compounds. However, efflux of linezolid, which is a hydrophilic drug, by EmrD-3 and other efflux pumps such as AcrAB, has changed this hypothesis [66,69]. The discovery of the role of EmrD-3 in linezolid resistance will lead to the identification of similar efflux pumps in Gram-positive bacteria. Currently no crystal or predicted 3D structures are available for EmrD-3.

## 5. LmrS from *Staphylococcus aureus*

LmrS is an efflux pump of the MFS family with 14 TMS identified in a clinically-isolated methicillin-resistant *Staphylococcus aureus* strain [70]. Unlike the QacA-family of plasmid-encoded efflux pumps, LmrS is encoded by a chromosomal gene. Proteins homologous to LmrS are widely distributed among the Gram-positive group of bacteria that includes *Staphylococcus*, *Enterococcus*, *Bacillus*, *Lactobacillus* and *Listeria.* The cloned *lmrS* gene conferred high antibiotic resistance to lincomycin, kanamycin, fusidic acid, linezolid, trimethoprim, florfenicol, chloramphenicol, erythromycin, streptomycin, fusidic acid, and kanamycin. The lincomycin resistance conferred by LmrS is further supported by an amino acid sequence similarity of 62% with the lincomycin resistance protein LmrB of *Bacillus subtilis.* Significantly, LmrS confers clinical levels of resistance to linezolid and fusidic acid, two important antimicrobials with strong activity against MRSA. Interestingly, the *lmrS* gene is present in both methicillin-resistant and -sensitive *S. aureus* strains. Thus, it needs to be determined if other regulatory factors play any role in the expression of *lmrS* in *S. aureus*. A preliminary study has shown constitutive expression of *lmrS* in clinical strains of *S. aureus* [70].

## 6. Mdt(A) from *Lactococcus lactis* and *L. garvieae*

The multiple drug transporter Mdt(A) is a plasmid-encoded efflux pump found in *Lactococcus lactis* [51]. The protein has 418 amino acids that fold into 12 TMS, and is a member of the MFS with some interesting structural differences [51]. Mdt(A) has two antiporter motifs (motif C) on TMS5 and TMS9, and also a putative ATP-binding site. The substrates for Mdt(A) include 14-, 15- and 16-membered macrolides, lincosamides, streptogramins and tetracyclines [51]. Recently, a multidrug-efflux pump EfmA of *Enterococcus faecium* has been reported to have very high sequence similarity (86%) with Mdt(A) [71]. However, it is not known if Mdt(A) is H^+^ dependent, though the addition of glucose resulted in efflux. However, protonophores such as CCCP did not inhibit the efflux activity [51]. The molecular mechanism and the structure-function relationship responsible for drug transport by Mdt(A) remains to be elucidated. A recent study describes the presence of a mutated Mdt(A) in *Lactococcus garvieae* that did not confer elevated resistance to erythromycin or tetracycline [48]. The mutations were Val154Phe and Ile296Val in TMS5 and TMS9 respectively, the two antiporter motifs (motif C) of Mdt(A) [48]. Unlike in *L. lactis*, Mdt(A) of *L. garvieae* is chromosomally encoded [48].

## 7. QacA and QacB from *Staphylococcus aureus*

QacA is encoded by plasmid-borne genes in multidrug resistant *Staphylococcus aureus* [72–74]. Subsequently, the *qacA* gene was found to be widespread among *Staphylococcus aureus* strains isolated from clinical environments [75]. The QacA efflux pump extrudes structurally diverse monovalent and divalent cationic substrates, the most prominent among them being the quaternary ammonium compounds or the Qacs [76,77]. QacA is 514 amino acids long, traverses the membrane 14 times and is energized by protons (H^+^) [78]. QacA was the first efflux protein of the MFS family with 14 TMS. QacB, which is also plasmid-encoded, confers resistance only to monovalent cationic substrates, confers little or no resistance to divalent cationic substrates, and the nucleotide sequence that encodes QacB differs from QacA only by seven nucleotides [79]. Structure-function analyses have demonstrated the functional importance of key amino acid residues in the transport of drug substrates by QacA, making these critical residues prime targets for drug design studies of putative efflux pump inhibitors [49,80–85]. The presence of acidic residues at amino acid positions 322 or 323 is essential for QacA or QacB to efflux divalent cations [79]. The expression of *qacA* genes is regulated by a transcription regulator, QacR, which belongs to the *tetR* family of regulators [86].

## 8. NorA, NorB and NorC from *Staphylococcus aureus*

NorA, the first chromosomally-encoded efflux pump identified in *Staphylococcus aureus* is made up of 388 amino acids, with 12 TMS [87,88]. The *norA* gene is present in all of the whole genome sequences of *Staphylococcus aureus* strains currently available in the GenBank. Initially, *norA* was thought to specifically efflux the quinolone drug norfloxacin, but subsequently was found to confer resistance to a number of antimicrobials, including chloramphenicol [75,85]. The expression of *norA* is regulated by *mgrA*, a member of a *marR* group of transcriptional regulators [89]. NorB and NorC are each made up of 462 amino acids, both efflux pumps are organized into 14 TMS and confer resistance to quinolones, such as ciprofloxacin, norfloxacin, and sparfloxacin [90,91].

## 9. Conclusions and Future Directions

Bacterial multi-drug efflux pumps constitute a major mechanism for conferring multi-drug resistance in pathogenic bacteria that cause infectious disease [76,92]. Multi-drug efflux pumps reside in the biological membrane and actively extrude antimicrobial agents from pathogenic bacterial cells [92], thus conferring multi-drug resistance. Thus, multi-drug efflux pumps reduce the efficacy of chemotherapy for infection caused by bacteria that harbor these pumps, resulting in a serious health concern [93,94]. In order for translational science efforts to come to fruition, it will become necessary to identify key targets within efflux pumps to make effective inhibitors, which can then be used in modulation chemotherapy [9,10]. Unfortunately, it is poorly understood how these multi-drug efflux pumps function in terms of the structure-function relationships. Thus, lack of a clear molecular analysis of the multi-drug efflux pumps prevents investigators from ultimately seeking potential efflux inhibitors, because they lack precise information regarding key molecular targets, such as amino acids that bestow drug transport. Study of the molecular biology of multi-drug efflux pumps would identify important amino acids that confer their active efflux function. Knowing these amino acid targets that confer activity from a molecular standpoint would identify critical amino acids that would serve, therefore, as important targets for potential inhibition of multi-drug efflux [9,10]. Thus, inhibition of the multi-drug efflux pumps would aid in potentially restoring the effectiveness of antimicrobial chemotherapy of infectious disease caused by bacteria that have multi-drug efflux pumps. Therefore, morbidity and mortality frequencies may be diminished, because of multi-drug efflux inhibition [9,10]. Study of the structure-function relationships of multi-drug efflux pumps is therefore needed, in order to determine the functional roles of key amino acids that convey drug efflux from pathogenic bacteria and make translational medicine possible.

## References

[1] Drews T.D., Temte J.L., Fox B.C. (2006). Community-associated methicillin-resistant *Staphylococcus aureus*: review of an emerging public health concern. Wis. Med. J.

[2] Mandal S., Mandal M.D., Pal N.K. (2011). Cholera: A great global concern. Asian Pac. J. Trop. Med.

[3] Walsh T.J., Standiford H.C., Reboli A.C., John J.F., Mulligan M.E., Ribner B.S., Montgomerie J.Z., Goetz M.B., Mayhall C.G., Rimland D. (1993). Randomized double-blinded trial of rifampin with either novobiocin or trimethoprim-sulfamethoxazole against methicillin-resistant *Staphylococcus aureus* colonization: Prevention of antimicrobial resistance and effect of host factors on outcome. Antimicrob. Agents Chemother.

[4] Walsh C.T., Fischbach M.A. (2009). New ways to squash superbugs. Sci. Am.

[5] Kitaoka M., Miyata S.T., Unterweger D., Pukatzki S. (2011). Antibiotic resistance mechanisms of *Vibrio cholerae*. J. Med. Microbiol.

[6] Prabaker K., Weinstein R.A. (2011). Trends in antimicrobial resistance in intensive care units in the United States. Curr. Opin. Crit. Care.

[7] Levy S.B. (1998). The challenge of antibiotic resistance. Sci. Am.

[8] Levy S.B. (2005). Antibiotic resistance-the problem intensifies. Adv. Drug Deliv. Rev.

[9] Lewis K. (1994). Multidrug resistance pumps in bacteria: Variations on a theme. Trends Biochem. Sci.

[10] Lewis K. (2001). In search of natural substrates and inhibitors of MDR pumps. J. Mol. Microbiol. Biotechnol.

[11] Wright G.D. (2007). The antibiotic resistome: The nexus of chemical and genetic diversity. Nat. Rev. Microbiol.

[12] Piddock L.J. (2006). Multidrug-resistance efflux pumps—Not just for resistance. Nat. Rev. Microbiol.

[13] Nikaido H. (1996). Multidrug efflux pumps of gram-negative bacteria. J. Bacteriol.

[14] Saier M.H., Paulsen I.T. (2001). Phylogeny of multidrug transporters. Semin. Cell Dev. Biol..

[15] Putman M., van Veen H.W., Konings W.N. (2000). Molecular properties of bacterial multidrug transporters. Microbiol. Mol. Biol. Rev.

[16] Li X.Z., Nikaido H. (2009). Efflux-mediated drug resistance in bacteria: An update. Drugs.

[17] Okusu H., Ma D., Nikaido H. (1996). AcrAB efflux pump plays a major role in the antibiotic resistance phenotype of *Escherichia coli* multiple-antibiotic-resistance (Mar) mutants. J. Bacteriol.

[18] Paulsen I.T., Skurray R.A., Tam R., Saier M.H., Turner R.J., Weiner J.H., Goldberg E.B., Grinius L.L. (1996). The SMR family: A novel family of multidrug efflux proteins involved with the efflux of lipophilic drugs. Mol. Microbiol..

[19] Morar M., Wright G.D. (2010). The genomic enzymology of antibiotic resistance. Annu. Rev. Genet.

[20] Paulsen I.T. (2003). Multidrug efflux pumps and resistance: Regulation and evolution. Curr. Opin. Microbiol.

[21] Cui J., Davidson A.L. (2011). ABC solute importers in bacteria. Essay. Biochem.

[22] Saier M.H., Beatty J.T., Goffeau A., Harley K.T., Heijne W.H., Huang S.C., Jack D.L., Jahn P.S., Lew K., Liu J. (1999). The major facilitator superfamily. J. Mol. Microbiol. Biotechnol..

[23] Nikaido H., Takatsuka Y. (2009). Mechanisms of RND multidrug efflux pumps. Biochim. Biophys. Acta.

[24] Griffith J.K., Baker M.E., Rouch D.A., Page M.G., Skurray R.A., Paulsen I.T., Chater K.F., Baldwin S.A., Henderson P.J. (1992). Membrane transport proteins: Implications of sequence comparisons. Curr. Opin. Cell Biol.

[25] He G.X., Thorpe C., Walsh D., Crow R., Chen H., Kumar S., Varela M.F. (2011). EmmdR, a new member of the MATE family of multidrug transporters, extrudes quinolones from *Enterobacter cloacae*. Arch. Microbiol.

[26] He G.X., Zhang C., Crow R.R., Thorpe C., Chen H., Kumar S., Tsuchiya T., Varela M.F. (2011). SugE, a new member of the SMR family of transporters, contributes to antimicrobial resistance in *Enterobacter cloacae*. Antimicrob. Agents Chemother.

[27] Henderson P.J., Roberts P.E., Martin G.E., Seamon K.B., Walmsley A.R., Rutherford N.G., Varela M.F., Griffith J.K. (1993). Homologous sugar-transport proteins in microbes and man. Biochem. Soc. Trans.

[28] Levy S.B., McMurry L. (1974). Detection of an inducible membrane protein associated with R-factor-mediated tetracycline resistance. Biochem. Biophys. Res. Commun.

[29] Levy S.B., McMurry L. (1978). Plasmid-determined tetracycline resistance involves new transport systems for tetracycline. Nature.

[30] Levy S.B. (1992). Active efflux mechanisms for antimicrobial resistance. Antimicrob. Agents Chemother.

[31] Varela M.F., Griffith J.K. (1993). Nucleotide and deduced protein sequences of the class D tetracycline resistance determinant: Relationship to other antimicrobial transport proteins. Antimicrob. Agents Chemother.

[32] Varela M.F., Sansom C.E., Griffith J.K. (1995). Mutational analysis and molecular modelling of an amino acid sequence motif conserved in antiporters but not symporters in a transporter superfamily. Mol. Membr. Biol.

[33] Levy S.B. (2002). Active efflux, a common mechanism for biocide and antibiotic resistance. Symp. Ser. Soc. Appl. Microbiol.

[34] Kaneko M., Yamaguchi A., Sawai T. (1985). Energetics of tetracycline efflux system encoded by Tn10 in *Escherichia coli*. FEBS Lett.

[35] Chopra I., Hawkey P.M., Hinton M. (1992). Tetracyclines, molecular and clinical aspects. J. Antimicrob. Chemother.

[36] Guay G.G., Tuckman M., McNicholas P., Rothstein D.M. (1993). The *tet(K)* gene from *Staphylococcus aureus* mediates the transport of potassium in *Escherichia coli*. J. Bacteriol.

[37] Schwarz S., Cardoso M., Wegener H.C. (1992). Nucleotide sequence and phylogeny of the tet(L) tetracycline resistance determinant encoded by plasmid pSTE1 from *Staphylococcus hyicus*. Antimicrob. Agents Chemother.

[38] Henderson P.J., Maiden M.C. (1990). Homologous sugar transport proteins in *Escherichia coli* and their relatives in both prokaryotes and eukaryotes. Philos. Trans. R. Soc. Lond. B Biol. Sci.

[39] Maiden M.C., Davis E.O., Baldwin S.A., Moore D.C., Henderson P.J. (1987). Mammalian and bacterial sugar transport proteins are homologous. Nature.

[40] Marger M.D., Saier M.H. (1993). A major superfamily of transmembrane facilitators that catalyse uniport, symport and antiport. Trends Biochem. Sci..

[41] Baldwin S.A., Henderson P.J. (1989). Homologies between sugar transporters from eukaryotes and prokaryotes. Annu. Rev. Physiol.

[42] Sheridan R.P., Chopra I. (1991). Origin of tetracycline efflux proteins: Conclusions from nucleotide sequence analysis. Mol. Microbiol.

[43] Lomovskaya O., Lewis K. (1992). Emr, an *Escherichia coli* locus for multidrug resistance. Proc. Natl. Acad. Sci. USA.

[44] Neyfakh A.A. (1992). The multidrug efflux transporter of *Bacillus subtilis* is a structural and functional homolog of the *Staphylococcus* NorA protein. Antimicrob. Agents Chemother.

[45] De Jesus M., Jin J., Guffanti A.A., Krulwich T.A. (2005). Importance of the GP dipeptide of the antiporter motif and other membrane-embedded proline and glycine residues in tetracycline efflux protein Tet(L). Biochemistry.

[46] Jin J., Guffanti A.A., Bechhofer D.H., Krulwich T.A. (2002). Tet(L) and tet(K) tetracycline-divalent metal/H^+^ antiporters: Characterization of multiple catalytic modes and a mutagenesis approach to differences in their efflux substrate and coupling ion preferences. J. Bacteriol.

[47] Pasrija R., Banerjee D., Prasad R. (2007). Structure and function analysis of CaMdr1p, a major facilitator superfamily antifungal efflux transporter protein of *Candida albicans*: Identification of amino acid residues critical for drug/H^+^ transport. Eukaryot. Cell.

[48] Walther C., Rossano A., Thomann A., Perreten V. (2008). Antibiotic resistance in *Lactococcus* species from bovine milk: Presence of a mutated multidrug transporter m*dt(A)* gene in susceptible *Lactococcus garvieae* strains. Vet. Microbiol.

[49] Hassan K.A., Galea M., Wu J., Mitchell B.A., Skurray R.A., Brown M.H. (2006). Functional effects of intramembranous proline substitutions in the staphylococcal multidrug transporter QacA. FEMS Microbiol. Lett.

[50] Chandrasekaran A., Ojeda A.M., Kolmakova N.G., Parsons S.M. (2006). Mutational and bioinformatics analysis of proline- and glycine-rich motifs in vesicular acetylcholine transporter. J. Neurochem.

[51] Perreten V., Schwarz F.V., Teuber M., Levy S.B. (2001). Mdt(A), a new efflux protein conferring multiple antibiotic resistance in *Lactococcus lactis* and *Escherichia coli*. Antimicrob. Agents Chemother.

[52] Ginn S.L., Brown M.H., Skurray R.A. (2000). The TetA(K) tetracycline/H^(+)^ antiporter from *Staphylococcus aureus*: Mutagenesis and functional analysis of motif C. J. Bacteriol.

[53] Saraceni-Richards C.A., Levy S.B. (2000). Second-site suppressor mutations of inactivating substitutions at gly247 of the tetracycline efflux protein, Tet(B). J. Bacteriol.

[54] Tamura N., Konishi S., Yamaguchi A. (2003). Mechanisms of drug/H^+^ antiport: Complete cysteine-scanning mutagenesis and the protein engineering approach. Curr. Opin. Chem. Biol.

[55] Nelson M.L., Levy S.B. (1999). Reversal of tetracycline resistance mediated by different bacterial tetracycline resistance determinants by an inhibitor of the Tet(B) antiport protein. Antimicrob. Agents Chemother.

[56] Nelson M.L., Park B.H., Andrews J.S., Georgian V.A., Thomas R.C., Levy S.B. (1993). Inhibition of the tetracycline efflux antiport protein by 13-thio-substituted 5-hydroxy-6-deoxytetracyclines. J. Med. Chem.

[57] Nelson M.L., Park B.H., Levy S.B. (1994). Molecular requirements for the inhibition of the tetracycline antiport protein and the effect of potent inhibitors on the growth of tetracycline-resistant bacteria. J. Med. Chem.

[58] Wright G.D., Sutherland A.D. (2007). New strategies for combating multidrug-resistant bacteria. Trends Mol. Med.

[59] Rahman M.M., Garvey M., Piddock L.J., Gibbons S. (2008). Antibacterial terpenes from the oleo-resin of *Commiphora molmol* (Engl.). Phytother. Res.

[60] Edgar R., Bibi E. (1999). A single membrane-embedded negative charge is critical for recognizing positively charged drugs by the *Escherichia coli* multidrug resistance protein MdfA. EMBO J.

[61] Adler J., Bibi E. (2002). Membrane topology of the multidrug transporter MdfA: Complementary gene fusion studies reveal a nonessential *C*-terminal domain. J. Bacteriol.

[62] Sigal N., Cohen-Karni D., Siemion S., Bibi E. (2006). MdfA from *Escherichia coli*, a model protein for studying secondary multidrug transport. J. Mol. Microbiol. Biotechnol.

[63] Begum A., Rahman M.M., Ogawa W., Mizushima T., Kuroda T., Tsuchiya T. (2005). Gene cloning and characterization of four MATE family multidrug efflux pumps from *Vibrio cholerae* non-O1. Microbiol. Immunol.

[64] Woolley R.C., Vediyappan G., Anderson M., Lackey M., Ramasubramanian B., Jiangping B., Borisova T., Colmer J.A., Hamood A.N., McVay C.S. (2005). Characterization of the *Vibrio cholerae vceCAB* multiple-drug resistance efflux operon in *Escherichia coli*. J. Bacteriol.

[65] Bina X.R., Provenzano D., Nguyen N., Bina J.E. (2008). *Vibrio cholerae* RND family efflux systems are required for antimicrobial resistance, optimal virulence factor production, and colonization of the infant mouse small intestine. Infect. Immun.

[66] Smith K.P., Kumar S., Varela M.F. (2009). Identification, cloning, and functional characterization of EmrD-3, a putative multidrug efflux pump of the major facilitator superfamily from *Vibrio cholerae* O395. Arch. Microbiol.

[67] Zurenko G.E., Yagi B.H., Schaadt R.D., Allison J.W., Kilburn J.O., Glickman S.E., Hutchinson D.K., Barbachyn M.R., Brickner S.J. (1996). *In vitro* activities of U-100592 and U-100766, novel oxazolidinone antibacterial agents. Antimicrob. Agents Chemother.

[68] Xiong L., Kloss P., Douthwaite S., Andersen N.M., Swaney S., Shinabarger D.L., Mankin A.S. (2000). Oxazolidinone resistance mutations in 23S rRNA of *Escherichia coli* reveal the central region of domain V as the primary site of drug action. J. Bacteriol.

[69] Bohnert J.A., Kern W.V. (2005). Selected arylpiperazines are capable of reversing multidrug resistance in *Escherichia coli* overexpressing RND efflux pumps. Antimicrob. Agents Chemother.

[70] Floyd J.L., Smith K.P., Kumar S.H., Floyd J.T., Varela M.F. (2010). LmrS is a multidrug efflux pump of the major facilitator superfamily from *Staphylococcus aureus*. Antimicrob. Agents Chemother.

[71] Nishioka T., Ogawa W., Kuroda T., Katsu T., Tsuchiya T. (2009). Gene cloning and characterization of EfmA, a multidrug efflux pump, from *Enterococcus faecium*. Biol. Pharm. Bull.

[72] Tennent J.M., Lyon B.R., Midgley M., Jones I.G., Purewal A.S., Skurray R.A. (1989). Physical and biochemical characterization of the *qacA* gene encoding antiseptic and disinfectant resistance in *Staphylococcus aureus*. J. Gen. Microbiol.

[73] Mitchell B.A., Brown M.H., Skurray R.A. (1998). QacA multidrug efflux pump from *Staphylococcus aureus*: Comparative analysis of resistance to diamidines, biguanidines, and guanylhydrazones. Antimicrob. Agents Chemother.

[74] Rouch D.A., Cram D.S., DiBerardino D., Littlejohn T.G., Skurray R.A. (1990). Efflux-mediated antiseptic resistance gene *qacA* from *Staphylococcus aureus*: Common ancestry with tetracycline- and sugar-transport proteins. Mol. Microbiol.

[75] Brown M.H., Skurray R.A. (2001). Staphylococcal multidrug efflux protein QacA. J. Mol. Microbiol. Biotechnol.

[76] Saidijam M., Benedetti G., Ren Q., Xu Z., Hoyle C.J., Palmer S.L., Ward A., Bettaney K.E., Szakonyi G., Meuller J. (2006). Microbial drug efflux proteins of the major facilitator superfamily. Curr. Drug Targets.

[77] Paulsen I.T., Lewis K. (2001). Microbial multidrug efflux: Introduction. J. Mol. Microbiol. Biotechnol.

[78] Mitchell B.A., Paulsen I.T., Brown M.H., Skurray R.A. (1999). Bioenergetics of the staphylococcal multidrug export protein QacA. Identification of distinct binding sites for monovalent and divalent cations. J. Biol. Chem.

[79] Paulsen I.T., Brown M.H., Littlejohn T.G., Mitchell B.A., Skurray R.A. (1996). Multidrug resistance proteins QacA and QacB from *Staphylococcus aureus*: Membrane topology and identification of residues involved in substrate specificity. Proc. Natl. Acad. Sci. USA.

[80] Jia B., Zhou T.Q., Huang A.L., Huang W.X. (2008). Role of TMS5: Staphylococcal multidrug-efflux protein QacA. Chin. Med. J. (Engl.).

[81] Hassan K.A., Skurray R.A., Brown M.H. (2007). Transmembrane helix 12 of the *Staphylococcus aureus* multidrug transporter QacA lines the bivalent cationic drug binding pocket. J. Bacteriol.

[82] Xu Z., O’Rourke B.A., Skurray R.A., Brown M.H. (2006). Role of transmembrane segment 10 in efflux mediated by the staphylococcal multidrug transport protein QacA. J. Biol. Chem.

[83] Wu J., Hassan K.A., Skurray R.A., Brown M.H. (2008). Functional analyses reveal an important role for tyrosine residues in the staphylococcal multidrug efflux protein QacA. BMC Microbiol.

[84] Hassan K.A., Souhani T., Skurray R.A., Brown M.H. (2008). Analysis of tryptophan residues in the staphylococcal multidrug transporter QacA reveals long-distance functional associations of residues on opposite sides of the membrane. J. Bacteriol.

[85] Hassan K.A., Skurray R.A., Brown M.H. (2007). Active export proteins mediating drug resistance in staphylococci. J. Mol. Microbiol. Biotechnol.

[86] Grkovic S., Brown M.H., Roberts N.J., Paulsen I.T., Skurray R.A. (1998). QacR is a repressor protein that regulates expression of the *Staphylococcus aureus* multidrug efflux pump QacA. J. Biol. Chem.

[87] Ubukata K., Itoh-Yamashita N., Konno M. (1989). Cloning and expression of the *norA* gene for fluoroquinolone resistance in *Staphylococcus aureus*. Antimicrob. Agents Chemother.

[88] Yoshida H., Bogaki M., Nakamura S., Ubukata K., Konno M. (1990). Nucleotide sequence and characterization of the *Staphylococcus aureus norA* gene, which confers resistance to quinolones. J. Bacteriol.

[89] Truong-Bolduc Q.C., Dunman P.M., Strahilevitz J., Projan S.J., Hooper D.C. (2005). MgrA is a multiple regulator of two new efflux pumps in *Staphylococcus aureus*. J. Bacteriol.

[90] Ding Y., Onodera Y., Lee J.C., Hooper D.C. (2008). NorB, an efflux pump in *Staphylococcus aureus* strain MW2, contributes to bacterial fitness in abscesses. J. Bacteriol.

[91] Hirai K., Aoyama H., Suzue S., Irikura T., Iyobe S., Mitsuhashi S. (1986). Isolation and characterization of norfloxacin-resistant mutants of *Escherichia coli* K-12. Antimicrob. Agents Chemother.

[92] Saidijam M., Bettaney K.E., Leng D., Ma P., Xu Z., Keen J.N., Rutherford N.G., Ward A., Henderson P.J., Szakonyi G. (2011). The MFS efflux proteins of gram-positive and gram-negative bacteria. Adv. Enzymol. Relat. Areas Mol. Biol.

[93] Gootz T.D. (2010). The global problem of antibiotic resistance. Crit. Rev. Immunol.

[94] Kumar A., Schweizer H.P. (2005). Bacterial resistance to antibiotics: Active efflux and reduced uptake. Adv. Drug Deliv. Rev.

